# Metabolite Profiles in Various Plant Organs of *Justicia gendarussa* Burm.f. and Its in Vitro Cultures

**DOI:** 10.3390/scipharm84030555

**Published:** 2016-04-13

**Authors:** Putu Indrayoni, Diah Intan Purwanti, Suwidji Wongso, Bambang E.W. Prajogo, Gunawan Indrayanto

**Affiliations:** 1 Faculty of Pharmacy, Airlangga University, Jl. Dharmawangsa Dalam, Surabaya 60286, Indonesia; indrayoniputu@gmail.com (P.I.); prajogo_ew@yahoo.com (B.E.W.P.); 2 PT. Angler BioChemLab, Plaza Graha Family C-25, Surabaya 60226, Indonesia; intan@anglerlab.net (D.I.P.); dir@anglerlab.net (S.W.)

**Keywords:** *Justicia gendarussa* Burm.f., UPLC-Qtof-MS, plant organs, in vitro cultures, PCA, secondary metabolites accumulation

## Abstract

Metabolite profiles of plant organs and their in vitro cultures of *Justicia gendarussa* have been studied by using Ultra Performance Liquid Chromatography-Quadrupole Time-of-Flight-Mass Spectrometry (UPLC-Qtof-MS). Samples of leaves, stems, roots, and shoot cultures showed similar patterns of metabolites, while samples of root cultures gave very different profiles. Concentrations of secondary metabolites in shoot cultures were relatively low compared to those in the leaves and roots of the plants. The results suggested that secondary metabolites in *J. gendarussa* were biosynthetized in the leaves, then transported to the roots.

## 1. Introduction

*Justicia gendarussa* Burm.f., an evergreen shrub belonging to the family Acanthaceae, is widely distributed in Indonesia, Sri Lanka, India, and Malaysia [1]. This plant has been reported for being cytotoxic [1], immunosuppressive [2], anti-inflammatory, analgesic [3], antioxidant, hepatoprotective [4,5], anti-anxiety [6], antibactericidal [7,8], anti-angiogenic [9], antifungal [10], antisickling [11], anthelmintic [12], larvicidal, adulticidal [13], and for inhibiting both HIV type 1 reverse transcriptase [14] and protein denaturation [15]. *n*-Butanol fraction of *J. gendarussa* leaves showed antifertility activity during in vitro and in vivo experiments; the main mechanism was through competitive and reversible inhibition of the spermatozoa hyaluronidase enzyme [16]. Pre-clinical and clinical trials have been conducted on the leaf extract of *J. gendarussa* in its development as an herbal drug [17,18,19,20,21].

Friedelin, β-sitosterol, lupeol, *O*-disubstituted aromatic amines, flavonoids, alkaloids, saponins, and phenolic compounds were previously identified in *J. gendarussa* [3,7,9,22,23]. 6,8-di-*C-α-*l-arabinosyl-apigenin, 6-*C-α-*l-arabinosyl-8-*C-β-*d-xylosyl-apigenin, and justidrusamides A-D were isolated from the leaves of *J. gendarussa* cultivated at Pacet, Indonesia [16,24]. A previous study has shown that metabolite profiles of *J. gendarussa* leaves derived from different locations in Indonesia were affected by their soil nutrients; the concentrations of Ca, P, and Cu in the soils could influence its metabolite profiles [25].

The variations in metabolites of different parts of the plant or its in vitro cultures have been reported in the literature for several plant species. Diosgenin was detected in plantlet and shoot cultures of *Costus speciosus,* but it could not be found in its callus cultures and root cultures [26]. Concentrations of phenolics, flavonoids, alkaloids, and phytosterols in callus cultures of *J. gendarussa* were equal or slightly increased compared to the original plants [27]. Ma and Gang reported that different tissues of turmeric possessed different metabolites profiles [28]. Accumulations of alkaloids in different organs of *Lycoris chinensis* were different [29]. Variations of secondary metabolites were observed in *Juniperus communis* [30].

For commercial production of herbal drugs, it is essential to determine where secondary metabolites are accumulated within a plant. The objective of the present study was to investigate the profiles of secondary metabolites in different plant organs of *J. gendarussa* and its in vitro cultures.

## 2. Materials and Methods

### 2.1. Materials and Chemicals

The *J. gendarussa* used in this work was of Papua origin and was planted at the campus of Airlangga University, Surabaya, Indonesia. This plant was identified by the Department of Pharmacognosy and Phytochemistry, Faculty of Pharmacy, Airlangga University (voucher no. 22/H3.1.5/DT/2013). Three plants (6 months old) derived from a single plant were cultivated in three different pots (plant 1, 2, and 3), and used as samples. Mature, dark green leaves and stems were collected 4–5 internodes from the terminal bud. Roots of 0–15 cm length were collected from the main trunk. Murashige Skoog (MS) media supplemented with 0.1 g·L^−1^ myo-inositol, 30 g·L^−1^ sucrose, and different hormone combinations were used for the in vitro cultures; media A: 6 mg·L^−1^ benzylaminopurine (BAP), media B: 6 mg·L^−1^ naphthaleneacetic acid (NAA), and media C: 6 mg·L^−1^ indolebutyric acid (IBA). Cultures were incubated under continuous light in a growth room illuminated with cool white fluorescent tubes (Philip Lifemax Cool Daylight TLD 36W/54-765) (Philip Lighting, Jakarta, Indonesia) at 25 ± 2 °C. The subculturing period was 21 days. Plant parts and in vitro cultures were air-dried (Loss on Drying were 1.1% ± 0.3%, *n* = 63) and powdered. Table 1 summarizes codes of the samples.

Methanol, ethanol, and formic acid (analytical reagent grade) were from Merck (Darmstadt, Germany). Purified water was from Sigma-Aldrich (St. Louis, MO, USA), acetic acid from J.T. Baker (Phillipsburg, NJ, USA), and NaOH from Agilent (Agilent solution for HPCE) (Mulgrave, Victoria, Australia). All samples were filtered through the 0.2 µm Agilent econo filter polyvinylidene difluoride (PVDF) 13 mm.

### 2.2. Preparation of Extracts and Quality Control (QC) Samples

All the samples of leaves, stems, roots and in vitro cultures of *J. gendarussa* (Table 1) were extracted in triplicate as described before [25]. The QC samples were prepared according to the published method [31].

### 2.3. Instrumentation

Samples were analyzed using the UPLC Dionex Ultimate 3000 RS LC (Dionex Suftron, GmbH, Thermo Fischer Scientific, Germening, Germany) coupled to the QTOF Bruker Maxis Impact HD (Bruker Daltonik, Bremen, Germany), equipped with an Enclosure services interface operating in negative ion mode. It had a mass range of *m*/*z* 50–1000, the capillary voltage was 2500 V, dry N_2_ gas flow of 8.0 L/min (200 °C), nebulizer pressure 2.0 bars, end plate offset 500 V, collision energy 25 eV, and an acquisition time factor of 1 s.

Chromatographic separation was carried out using an Acclaim RSLC 120 C18 column (2.2 µm, 120 Å, 2.1 × 100 mm) (Dionex, Thermo Fischer Scientific, Sunnyvale, CA, USA). The mobile phase consisted of 90% methanol with 5 mM ammonium acetate and 50% methanol with 5 mM ammonium acetate. Injection volume was 1.0 µL. Mass calibration was performed using 1 mM sodium formate/acetate in 50% isopropanol with 0.2% formic acid, HCOO(NaCOOH)_1_ (*m*/*z* 112.9856), Ac(NaAc)_1_ (*m*/*z* 141.0169), and Ac(NaF)_1_ (*m*/*z* 127.0013).

Data analysis and calculations were performed using the following software: Data Analysis 4.1 (SmartFormula, SmartFormula 3D, Isotope Pattern, and Fragmentation Explorer), Profile Analysis 2.1 (PCA and Bucket Statistic Plot), Metabolite Detect 2.0 from Bruker Daltonik, Bremen, Germany, MetFrag (version 2010) [32], Metlin [33], and MassBank [34].

### 2.4. Data Processing

Automatic time alignment was performed on retention time (RT)-*m*/*z* pairs of 0.4 to 20 min. Data were grouped automatically into buckets with RT-*m*/*z* pairs of 0.5035 min and *m*/*z* 30.3587; the mass range was 200-700 Da with a mass tolerance 0.05 Da, normalized with the sum of bucket values, *pareto-*scaled, and a bucket filter of 2% as described before [25].

The proposed molecular formula was performed using SmartFormula based on the exact mass and isotopic pattern; the proposed fragmentation of the compound was generated using SmartFormula 3D. Then, the fragmentation pattern of the compounds were generated using MetFrag [32] and Fragmentation Explorer.

### 2.5. Analytical Method Validation

Stability testing and method validation (intra-day variability) were performed by injecting sample SC2A at different times: 0 h, 12 h, 18 h, and 24 h in triplicate. Principal component analysis (PCA) confirmed that the extracts were stable for at least 24 h, and showed acceptable intra- and inter-day variability.

For checking the reliability of the method for each series of experiments, the QC sample was injected three times at the beginning of the analysis, then regularly every 6–7 samples. Coefficient variations (CV) of the data set were evaluated according to the published method [31]. Our data showed >85.75% of the bucket data that showed the CV <30%. PCA models were cross-validated with full cross-validation and showed no outliers. The tight clustering of the QC samples in the PCA analysis showed the reliability of the method.

## 3. Results and Discussion

**Figure 1:**
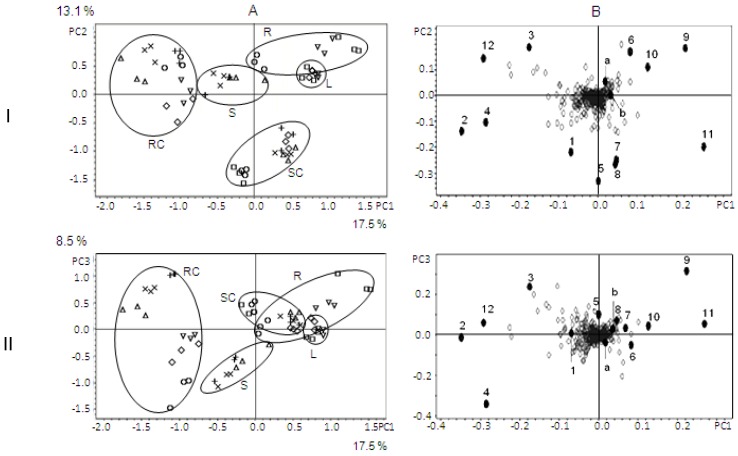
PCA score plots (**A**) and loading plots (**B**). Numbers (1–12) and lower case letters (a, b) refer to metabolites as listed in Table 2 and Table 3 and Figure 2. RC: root cultures, R: roots, SC: shoot cultures, S: shoots, L: leaves. I: PC1 versus PC2; II: PC1 versus PC3.

PCA analysis of pairs RT and *m*/*z* (Figure 1) showed definite discrimination of samples leaves (L), roots (R), stems (S), shoot cultures (SC), and samples of root cultures (RC). Samples L, R, and SC were not well-separated. The total explained variant for the three principle components (PC) were 39.1%. Score plots constructed by using more PCs (up to PC 8) showed similar discrimination patterns (the total explained variants PC 1 to PC 8 was 62.4%).

**Figure 2:**
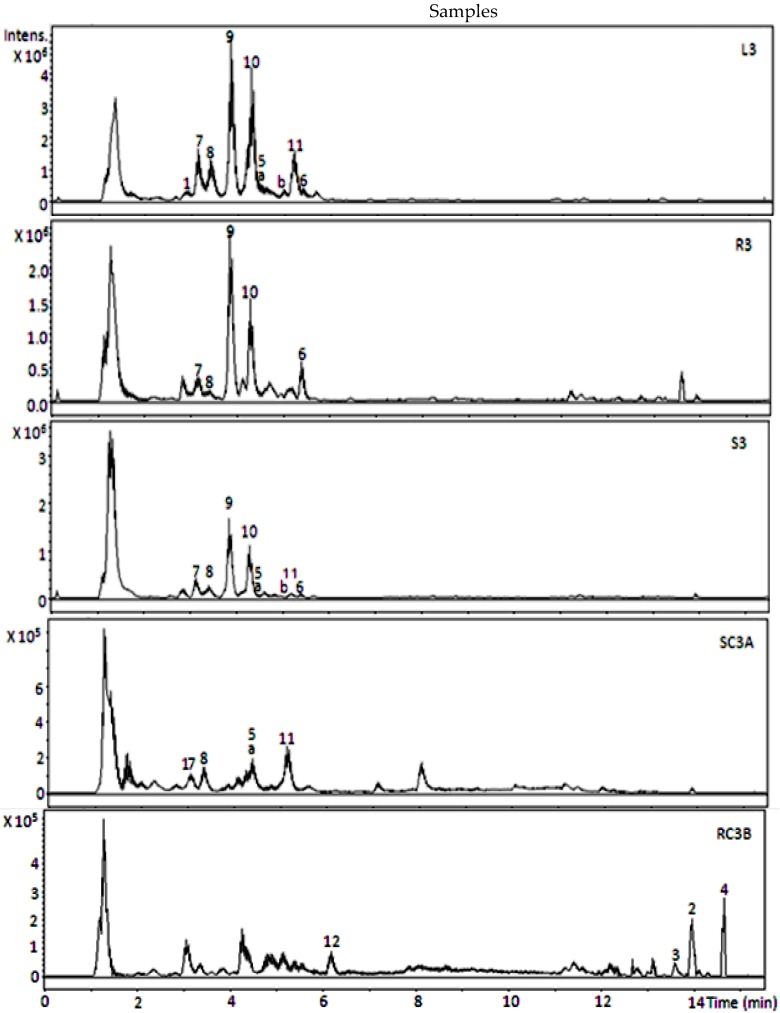
Total ion chromatogram (TIC) of selected samples. Numbers (1–12) and lower case letters (a, b) refer to metabolites as listed in Table 2 and Table 3.

The PCA score plot revealed that different combinations of plant growth hormones (media B and C) have relatively no influence on metabolite profiles of the root cultures. PCA score plots showed closeness among the cluster of metabolite profiles of leaves, roots, stems, and shoot cultures, while root cultures produced a very different metabolite profile. These were confirmed by their total ion chromatogram (TIC) patterns (Figure 2). TICs of leaves, roots, stems, and shoot cultures showed almost similar patterns, but root cultures yielded a distinctive profile. Relative intensities of the metabolites as shown by their TICs were confirmed with their bucket statistic plots. TICs also showed that the concentration of metabolites in the shoot cultures was relatively low compared to the leaves and roots of the plants.

The results suggest that secondary metabolites in *J. gendarussa* are biosynthesized in the leaves and then transported to the stems and roots.

### Identification of Metabolites

**Figure 3:**
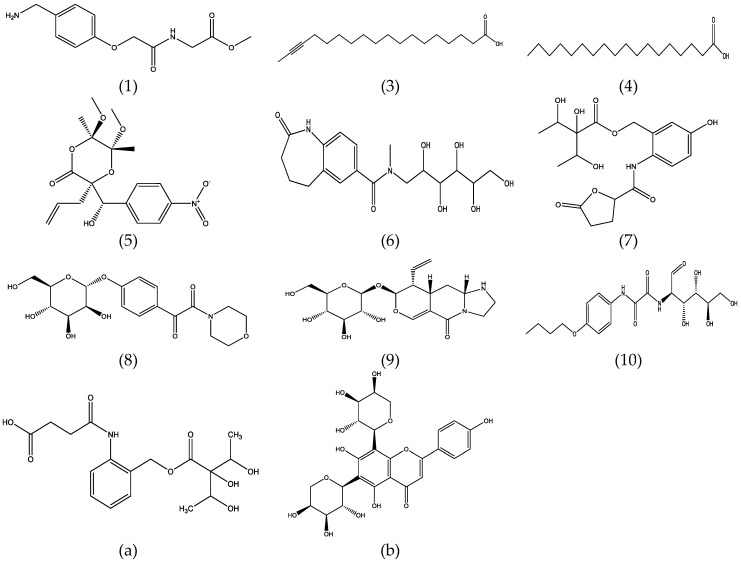
Chemical structures of the proposed metabolites.

Loading plots (Figure 1) showed that 12 significant metabolites (1–12) affected the clustering of the samples. Proposed metabolites and their fragmentation patterns are shown in Table 2 and Table 3 and Figure 3.

Metabolites **9** and **10** gave the highest intensity in leaves, roots, and stem samples. The presence of metabolites **a** and **b** in leaves have previously been reported [16,24,25]. Metabolites 3 and 4 were proposed as fatty acids; stearic acid and 9,12-octadecadienoic acid (Z, Z) have been reported to come from the methanolic extract of *J. wynaadensis* analyzed by gas chromatography–mass spectrometry (GC-MS) [35]. Metabolite **6** was proposed as protoberberine alkaloid; different protoberberine alkaloids were previously isolated from aerial parts of *Gendarussa vulgaris* Nees (synonym of *J. gendarussa*) [36].

Metabolite **7** was aminobenzyl alcohol derivate, and 2-aminobenzyl alcohol derivates were previously reported in the leaves of *J. gendarussa* [24,25,37]. Metabolite 8 was identified previously in *J. gendarussa* [25]. Chemical structures of compounds **2**, **11**, and **12** could not be matched to any within the database MetFrag [32], Metlin [33], and MassBank [34].

In conclusion, this present work has shown that metabolite profiles in the roots and leaves of *J. gendarussa* are almost identical, but the concentrations of metabolites in shoot cultures seemed very low compared to the leaves. Therefore, it is suggested to use leaves of *J. gendarussa* as the source for herbal drug raw materials; it seems that the application of tissue cultures as an alternative source for herbal drug production of *J. gendarussa* is not recommended.

## Figures and Tables

**Table 1:** Codes of the samples.

Samples	Codes
Leaves	L1, L2, L3
Roots	R1, R2, R3
Stems	S1, S2, S3
Shoot Cultures ^a^	SC1A, SC2A, SC3A
Root Cultures ^b^	RC1B, RC2B, RC3B
Root Cultures ^c^	RC1C, RC2C, RC3C

^a^ Cultivated on media A; ^b^ Cultivated on media B; ^c^ Cultivated on media C. Numbers (1–3) showed the plant origin.

**Table 2:** Proposed metabolites and their probable elemental formulas.

Metabolites	RT (min)	Ions	Measured *m*/*z*	Score (Err[mDa]; mSigma) Ions ^a^	Probable Elemental Formulas ^a,b^
			HRMS Ions		
			(*m*/*z* calc.) ^a^		
1	3.09	[M − H]^−^	251.1039	77	C_12_H_16_N_2_O_4_
			(251.1037)	(0.1;2.8)	
2	13.94	[M − H]^−^	255.2326	88	C_16_H_32_O_2_
			(255.2330)	(−0.3;1.7)	
3	13.64	[M − H]^−^	279.2339	63	C_18_H_32_O_2_
			(279.2330)	(1.0;12.9)	
4	14.66	[M − H]^−^	283.2638	85	C_18_H_36_O_2_
			(283.2643)	(−0.4;0.7)	
5	4.43	[M − H]^−^	380.1339	75	C_18_H_23_NO_8_
			(380.1351)	(−1.2;8.7)	
6	5.42	[M − H]^−^	381.1668	100	C_18_H_26_N_2_O_7_
			(381.1667)	(−0.1;5.6)	
		[2M − H]^−^	763.3405	96	C_36_H_52_N_4_O_14_
			(763.3407)	(−0.3;31.8)	
7	3.09	[M − H]^−^	396.1291	84	C_18_H_23_NO_9_
			(396.1300)	(−0.9;7.7)	
8	3.36	[M − H]^−^	396.1299	100	C_18_H_23_NO_9_
			(396.1300)	(0.1;6.4)	
9	3.84	[M − H]^−^	397.1611	89	C_18_H_26_N_2_O_8_
			(397.1616)	(−0.5;2.4)	
10	4.29	[M − H]^−^	397.1614	100	C_18_H_26_N_2_O_8_
			(397.1616)	(0.3;1.9)	
		[2M − H]^−^	795.3290	48	C_36_H_52_N_4_O_16_
			(795.3306)	(1.5;16.3)	
11	5.18	[M − H]^−^	533.1311	100	C_26_H_22_N_4_O_9_
			(533.1314)	(−0.3;6.3)	
12	6.17	[M − H]^−^	651.2301	100	C_31_H_40_O_15_
			(651.2294)	(0.7;10.0)	
a	4.26	[M − H]^−^	368.1357	69	C_17_H_23_NO_8_
			(368.1351)	(0.6;5.9)	
		[2M − H]^−^	737.2779	100	C_34_H_46_N_2_O_16_
			(737.2775)	(0.4;2.1)	
b	5.01	[M − H]^−^	533.1313	67	C_25_H_26_O_13_
			(533.1301)	(−1.3;5.0)	

^a^ Data were obtained using Smart Formula 3D; ^b^ Elemental formulas were confirmed with their isotope patterns. RT: retention time; HRMS: High Resolution Mass Spectrometry.

**Table 3:** Proposed metabolites and their fragmentations.

Metabolites	Score (Chem Spider ^a^/Pub Chem ^b^)	Explained ^c^ and MS/MS Fragment Ions ^d^	Measured *m*/*z* HRMS Fragment Ions	Score(Err[mDa]; mSigma) Fragment Ions ^d^	Proposed Metabolites	Metabolite IDs and References
			(*m*/*z* calc.) ^d^			
**1**	0.991/1.0	[C_11_H_12_NO_4_]^−^	222.0778	55	Methyl N-{[4-(aminomethyl)phenoxy]acetyl} glycinate ^c^	ID 11862597 ^a^; CID 16777361 ^b^;
			(222.0772)	(0.6;21.8)		
		[C_5_H_8_NO_4_]^−^	146.0463	75		
			(146.0459)	(0.4;10.8)		
		[C_5_H_6_NO_3_]^−^	128.0355	100		
			(128.0353)	(0.1;1.8)		
		[C_7_H_8_NO]^−^	122.0612	66		
			(122.0611)	(0.1;4.3)		
		[C_7_H_6_NO]^−^	120.0451	59		
			(120.0455)	(−0.4;3.8)		
		[C_3_H_5_O_2_]^−^	73.0294	63		
			(73.0295)	(−0.1;6.4)		
**2**	-/-	[C_11_H_15_O]^−^	163.1125	43	Unknown ^g^	
			(163.1128)	(0.3;21.8)		
**3**	1.0/1.0	[C_15_H_29_O_2_]^−^	241.2177	45	16-Octadecynoic acid ^c,e^	ID 4472124 ^a^; CID 5312699 ^b^; ID 74231 e
			(241.2173)	(−0.4;11.1)		
		[C_14_H_27_O_2_]^−^	227.2024	24		
			(227.2017)	(0.7;33.5)		
**4**	0.911/0.911	[C_16_H_31_O_2_]^−^	255.2326	42	Stearic acid ^c,e^	ID 5091 ^a^; CID 164708 ^b^; ID 189 ^e^; C01530 ^f^
			(255.2330)	(0.4;17.2)		
**5**	1.0/0.835	[C_12_H_10_NO_4_]^−^	232.0611	69	(3S,5R,6R)-3-Allyl-3-[(S)-hydroxy(4-nitrophenyl) methyl]-5,6-dimethoxy-5,6-dimethyl-1,4-dioxan-2-one ^c^	ID 9328344 ^a^; CID 11153236 ^b^
			(232.0615)	(0.4;13.5)		
		[C_11_H_10_NO_3_]^−^	204.0654	26		
			(204.0666)	(1.2;36.7)		
		[C_6_H_11_O_4_]^−^	147.0658	51		
			(147.0663)	(−0.5;6.0)		
**6**	0.968/-	[C_6_H_11_O_5_]^−^	163.0609	64	1-Deoxy-1-{methyl[(2-oxo-2,3,4,5-tetrahydro-1H-1-benzazepin-7-yl) carbonyl] amino}hexitol ^c^	ID 34743441 ^a^
			(163.0612)	(0.3;6.2)		
		[C_6_H_11_O_4_]^−^	147.0663	75		
			(147.0663)	(0.0;4.9)		
**7**	1.0/-	[C_6_H_11_O_5_]^−^	163.0610	89	1,5-Dideoxy-3-C-{[(5-hydroxy-2-{[(5-oxotetra hydro-2-furanyl) carbonyl] amino}benzyl) oxy]carbonyl} pentitol ^c^	ID 29814435 ^a^ [25]
			(163.0612)	(0.1;1.5)		
**8**	0.802/-	[C_6_H_11_O_5_]^−^	163.0611	85	4-[4-Morpholinyl(oxo)acetyl] phenyl α-d-manno pyranoside ^c^	ID 32768629 ^a^ [25]
			(163.0612)	(−0.1;2.5)		
		[C_4_H_5_O_3_]^−^	101.0241	50		
			(101.0244)	(0.3;5.0)		
**9**	0.841/-	[C_6_H_11_O_5_]^−^	163.0612	84	(8S,9R,9aS, 10aR)-5-Oxo-9-vinyl-1,2,3,8,9,9a,10,10a-octahydro-5H-imidazo[1,2-a] pyrano [4,3-d] pyridin-8-yl β-d-glucopyranoside ^c^	ID 26570736 ^a^
			(163.0612)	(−0.0;5.2)		
		[C_5_H_9_O_5_]^−^	149.0452	77		
			(149.0455)	(0.3;3.5)		
		[C_4_H_7_O_4_]^−^	119.0349	35		
			(119.0350)	(0.1;26.5)		
		[C_4_H_5_O_3_]^−^	101.0246	84		
			(101.0244)	(0.2;1.4)		
**10**	0.885/0.97	[C_6_H_11_O_5_]^−^	163.0612	86	2-({[(4-Butoxyphenyl)amino](oxo)acetyl}amino)-2-deoxy-d-glucose ^c^	ID 21249273 ^a^; CID 24838413 ^b^
			(163.0612)	(−0.0;3.0)		
		[C_4_H_7_O_4_]^−^	119.0350	86		
			(119.0350)	(−0.1;2.9)		
		[C_4_H_5_O_3_]^−^	101.0244	52		
			(101.0244)	(0.1;6.4)		
**11**	-/-	[C_6_H_11_O_4_]^−^	147.0663	96	Unknown ^g^	
			(147.0663)	(0.0;1.7)		
**12**	-/-	[C_27_H_32_O_7_]^−^	468.2158	66	Unknown ^g^	
			(468.2154)	(0.4;20.1)		
**a**	1.0/1.0	[C_6_H_11_O_5_]^−^	163,0618	64	3-C-[({2-[(3-Carboxypropanoyl)amino]benzyl}oxy)carbonyl]-1,5-dideoxy-L-arabinitol(Justidrusamide A/B) ^c^	ID 22943323 ^a^; CID 38352741^b^; [24,25]
			(163,0612)	(0.6;5.9)		
		[C_4_H_5_O_3_]^−^	101,0246	81		
			(101,0244)	(−0.2;2.5)		
**b**	0.969/1.0	[C_9_H_5_O_2_]^−^	145,0301	52	6,8-Di-C-alpha-l-arabino pyranosylapigenin ^c,e^	ID 26504074 ^a^; CID 10918510 ^b^; ID 48669 ^e^; [16,25]
			(145,0295)	(0.6;24.1)		
		[C_4_H_5_O_3_]^−^	101.0249	50		
			(101.0244)	(−0.5;8.8)		
		89.0249	52			
		(89.0244)	(−0.5;6.7)			

Data were obtained using: ^a^ MetFrag (ChemSpider); ^b^ MetFrag (PubChem); ^c^ MetFrag; ^d^ Smart Formula 3D; ^e^ Metlin; ^f^ MetFrag (KEGG); ^g^ No match resulted from MetFrag, Metlin, and MassBank. MS/MS: Mass Spectrometry/Mass Spectrometry.
